# Phytochemical and pharmacological aspects of *Descurainia sophia* Webb ex Prantl: modern and traditional applications

**Published:** 2016

**Authors:** Majid Nimrouzi, Mohammad Mahdi Zarshenas

**Affiliations:** 1 *Research Institute for Islamic and Complementary Medicine, Iran University of Medical Sciences, Tehran, Iran*; 2 *Essence of Parsiyan Wisdom Institute, Traditional Medicine and Medicinal Plant Incubator, Shiraz University of Medical Sciences, Shiraz, Iran*; 3 *Medicinal Plants Processing Research Center, Shiraz University of Medical Sciences, Shiraz, Iran *; 4 *Department of Phytopharmaceuticals (Traditional Pharmacy), School of Pharmacy, Shiraz University of Medical Sciences, Shiraz, Iran*

**Keywords:** *Descurainia sophia*, Webb ex Prantl, *Pharmacology*, *Phytochemistry*, *Traditional medicine*

## Abstract

Seed of *Descurainia sophia * Webb ex Prantl has been traditionally prescribed as treatment for palpitation, varicose vein, varicocele, constipation, hemorrhoid, skin eruptions, and impotence. To outline a view for further approaches, current work compiled a survey on all relevant clinical properties of this medicament in addition to the traditional reports. To do this, databases as PubMed, Scopus, EMBASE, IranMedex and Science information databases (SID) were searched by keywords, i.e., “*Descurainia sophia*”, “*Khaksheer*”, and “Flixweed” as well as “pharmacology” and “phytochemistry”. According to the findings, scant experimental evaluation and clinical assessment have been performed on this medicament. Of those, only anti-inflammatory, analgesic, and antipyretic effects as well as antioxidant and anthelmintic activities were assessed and confirmed in experimental studies. Despite broad administration of this herb in folk and traditional medicine, only two human clinical trials in bowel discomfort and pregnant subjects were conducted. Taken as a whole, more comprehensive clinical evaluations should be conducted on respective applications to support those traditional and folk uses.

## Introduction

There are numerous herbal medicines reported in the Traditional Persian Medicine (TPM) which has not yet been evaluated for efficacy and safety. *Descurainia sophia* Webb ex Prantl (DS) seed, also known as flixweed, a commonly used herbal medicine in Iranian folk medicine is one of those medicinal herbs with prevalent use. Known as the largest traditional pharmacopeia of Persian medicine (Zarshenas et al., 2013 ▶), *Makhzan al-adviyah *(the storehouse of medicaments) has cited the herb as *Khobbah *(Khorasani, 2001 ▶). It is called *Khaksheer* among folks (Hossaini-Tabib, 1959 ▶). Few studies have shown the medical efficacies and safety of DS. Of those studies, anthelmintic, antioxidant and radical scavenging, analgesic, and anti-inflammatory as well as antipyretic effects have been experimentally assessed. In this regard, current study aimed to compile all proved clinical and pharmacological properties of DS as well as those effects cited in Persian medical and pharmaceutical manuscripts to outline a viewpoint for further research. 

## Materials and Methods

We searched databases of PubMed, Scopus, EMBASE, IranMedex, and SID to find the relevant clinical trials on medical properties of DS as well as those literatures dealt with the active ingredients of *Descurainia sophia* Webb ex Prantl. Keywords for this research were “*Descurainia sophia*”, “pharmacology” and “phytochemistry”. Data gathering from databases, i.e., PubMed, Scopus, EMBASE, IranMedex, and science information databases (SID) was performed up to 31^st^ Jan 2014. We also reviewed the TPM resources, including *Makhzan-al-Advieh* (The storehouse of medicaments), *Tohfatol-momenin *(Gift for the faithful), and *Raaz-e-Darman* (Secret of treatment) to find the medical benefits, considerations, and properties of DS seed (*Khobbah*) in TPM. Two human studies and also two experimental animal models were found and reviewed. 

**Limitations**

There are few studies on the therapeutic effects of DS; however, it is a commonly used herb in folk medicine. We could not find relevant randomized clinical trials and systematic reviews to enrich our study. *Khobbah* or flixweed is scarcely cited in the medieval TPM resources such as Canon of Avicenna and Continents of Rhazes. Hence, we used more recent resources from early 17^th^ up to 20^th^ century, including *Makhzan-al Advieh* of Aghili, *Tohfat-al-Momenin* of Hakim Mommen, and *Raz-e-Darman* of Ahmadieh. 

## Results

**Description and phytochemistry of** ***Descurainia sophia*** **Webb ex Prantl**

*Descurainia sophia * Webb ex Prantlor *Sisymbrium sophia* with common name of Flixweed belongs to the family Brassicaceae. In traditional Persian manuscripts, it is cited as *Khaksheer, Khakshee*, *Todri*, *Khobbah *and *Bazr-al-khomkhom *(Mirheidar, 2005 ▶). The plant is widely distributed in the different parts of the world (Peng et al., 1997 ▶). It is a weed with high germination, especially in late autumn and early spring (Li et al., 2005 ▶). DS is also distributed in most parts of Iran. It is an annual plant which grows up to nearly 80 cm high. DS has pale yellow flowers arranged in terminal clusters (Mirheidar, 2005 ▶). 

Using Soxhlet extraction, major fatty acid composition yielded from the leaves, stems, and roots were oleic acid, linolenic acid, linoleic acid, and palmitic acid. Polyunsaturated fatty acid constituents were found to be higher than saturated ones (Tavakoli et al., 2012 ▶). The extracted oil from DS seed contains fatty acids such as oleic acid, erucic acid, linolenic acid, linoleic acid, palmitic acid, and stearic acid. GC/MS analysis of DS aerial parts revealed that monoterpenes, sesquiterpenes, and their derivatives were the main ingredients and in detail resulted in the appearance of cis-β-ocimene (20.1%), menthol (11.27%), neoisomenthyl acetate (3.5%), alloaromadendrene (2.28%), and longicyclene (2.25%) as major constituents (Li et al., 2010 ▶). The volatile oils of DS seed contain benzyl, allyl, propenyl-isothiocyanate, and allyl disulfide constituents (Mirheidar, 2005 ▶). Some cardiac glycosides, flavonoids, and phenols are also isolated from DS seeds (Sun et al., 2004 ▶). Sun et al. isolated and purified new nor-lignan constituents, aryldihydronaphthoic acid, and descuraicacid from the seeds (Sun et al., 2006 ▶). Descurainoside (a new sulphur glycoside), descurainin A, descurainoside B, descurainolide A and B (two new lactones), descurainin, and sinapic acid were also isolated from the seeds (Sun et al., 2004 ▶; Sun et al., 2005 ▶). Wang et al. isolated and reported three new quercetin compounds from DS for the first time (Wang et al., 2004 ▶). Coumarin compounds including scopoletine, scopoline, xanthtoxol, isoscopoline, psoralene, xanthtoxin, and bergaptane as well as flavonoids namely quercetine, kaempferol, and isorhamnetine were also isolated and identified from DS aerial part (Mohamed and Mahrous, 2009 ▶). On the other side, by using silica gel column chromatography and employing the spectral data, β-sitosterol, daucosterol, sinapic acid, 4-hydroxy-3,5-dimethoxybenzaldehyde, kaempferol, sinapine bisulfate, quercetin, and quercetin-7-O-β-D-glucopyranosyl (1,6)-β-D-glucopyranoside were isolated. Of these, the last compound was found to be new (Kai and Xian, 2003 ▶). Using flame atomic absorption spectrometry (FAAS), some DS trace elements in rich content were also identified. These elements were determined as iron, zinc, manganese, and copper with 136.50, 39.76, 29.51 and 8.11 μg/g, respectively (Zhongfeng, 2007 ▶).

**Experimental, animal, and clinical studies**

Maraghi et al. showed that the hydroalcoholic extract of DS possessed therapeutic effects on mice infected with *Himeonolepis nana *(Maraghi and Torfi, 2002 ▶). Ayyubi et al. proved that the aqueous extract of the seed can relieve the castor oil-induced diarrhea in rats (Ayoobi et al., 2013 ▶). Alcoholic extract of DS showed antipyretic, anti-inflammatory, and analgesic effects. Results of that study showed that the antipyretic effects of DS extract in 400 mg/kg were nearly the same as diclofenac Na in hyperthermic rats. Regarding anti-inflammatory activity, hydroalcoholic extract of DS decreased paw thickness for more than 3 hours which could be due to the coumarins. DS extract possessed 40% protection against writhing compared with control and paracetamol (Mohamed and Mahrous, 2009 ▶). Mirzaei et al. showed the antioxidant effects of the hydroalchoholic extract of DS in vitro. The total phenol content of DS extract was determined as 94 ± 12.1 mg galic acid/g dry material. On the other hand, free-radical scavenging and antioxidant activity assessments of DS were carried out using 1,1-Diphenyl-2-picrylhydrazyl (DPPH), 2,2'-azinobis 3-ethylbenzothiazoline-6-sulphonic acid (ABTS), ferric reducing antioxidant power assay (FRAP), phosphomolybdenum (PMB), and reducing power (RP) methods (Mirzaei et al., 2011 ▶). That investigation reported the activities of DS extract as 1686 ± 16.3 μmol Trolox/g extract via DPPH method, 302 ± 6.6 μmol Trolox/g extract using ABTS assay, 624/37 ± 7.57 μmol Fe/g extract via FRAP method, 348.3 ± 17.6 μmol Trolox/g extract by PMB method, and 0.44 ± 0.05 via RP assay. Lee et al. found the potent cytotoxicity of cardenolide glycoside, the main active constituent derived from DS plant, against human cancer cells (Lee et al., 2013 ▶).

Pasalar et al. found that a combination of flixweed and prune can improve the frequency of bowel movement, ease of defecation, and feeling of fullness in Hajj pilgrims (Pasalar et al., 2013 ▶). Results of a human study proved that oral drink of DS seed in the last month of pregnancy helped ripening of cervix and facilitated vaginal delivery (Mohammadinia et al., 2012 ▶). 

**DS in the traditional medicine**

DS is a commonly used medicinal herb in China and India. DS seed is used as an herbal medicine to manage cough and prevent asthma in traditional Chinese medicine (TCM). It is also traditionally recommended as a cardiotonic herb (Sun et al., 2004 ▶). It is reported as a purgative, febrifuge, antipruritic, antihelmintic, expectorant, astringent, and litholytic agent (Mirheidar, 2005 ▶).

According to the humoral concepts of TPM resources, DS seed has warm and wet nature. It means that drinking the syrup of DS seed can make the body warmer and also increases the humidity of the gastrointestinal tracts (Ahmadieh, 2007 ▶; Khorasani, 2001 ▶). DS seeds, containing a number of nutraceuticals, are commonly used as a drinking syrup sample in TPM from long time ago. Despite the medicinal advantages of DS seed, preparation of a pharmaceutical syrup dosage form is a challenge regarding fast precipitation of DS seeds. Behbahani et al. used Persian gum and Tragacantha to physically stabilize the DS seed syrup (Behbahani and Abbasi, 2013 ▶). 

In TPM, DS seed is considered as a herbal medicine with warm and wet nature. Persian practitioners reported that the seeds can be *mobahhi* (aphrodisiac), *moshahhi* (appetizing), and *moghavvi* (digestive tonic) (Hossaini-Tabib, 1959 ▶; Khorasani, 2001 ▶). In Iranian folk medicine, the cold syrup of DS with sugar and chicory are used for cooling, and hot syrup of DS seed with sugar are administered as a laxative (Akbari et al., 2011 ▶). 

DS seed was traditionally considered as an effective remedy for the evacuation of the morbid *soada* (black bile) from the body in TPM. Accordingly, it was used in diseases resulting from the abundance of black bile in the body. These disorders include but not are limited to palpitation, dyspepsia, varicose vein, hematologic diseases, varicocele, constipation, and hemorrhoid (Ahmadieh, 2007 ▶). In TPM, DS seed is also prescribed for skin darkness, urticaria, eruptions in measles, hoarseness, and impotence (Mirheidar, 2005 ▶; Khorasani, 2001 ▶).

The most common undesirable effect traditionally reported from DS seeds was headache. Thus, it was recommended that tragacantha should be accompanied with DS to prevent that effect (Hossaini-Tabib, 1959 ▶; Khorasani, 2001 ▶).

## Discussion

Concerning the presence of different classes of secondary metabolites and compounds in aerial part and seeds of DS, the plant has demonstrated numerous pharmacological properties (Table 1). 

The anti-inflammatory, antipyretic, and analgesic activities are generally related to the presence of phenolic compounds (Mohamed and Mahrous, 2009 ▶). DS has also been reported to have anticancer activity. Mechanism underlying this activity is reported to be the suppression in cancer cell growth as well as the inhibition of the chronic inflammation (Lee et al., 2013 ▶). Cardiotonic effect of DS seed was cited both in TCM and TPM. The cardiac glycosides which have been isolated from DS seeds may contribute to those therapeutic effects. DS acts like a stool softener and bowel smooth muscle relaxant. The mucilage produced in a macerated preparation of DS seed in water absorbs the water in the bowel lumen and softens the stool. The allyl disulfide compound in DS seed most likely acts as a smooth muscle relaxant and may facilitate the defecation (Sun et al., 2005 ▶). According to an investigation by Ayyubi et al., DS could reduce the frequency of defecation. That study denoted that phenolic compounds in DS seeds are responsible for this property.

Studies have revealed the safety of DS (LD_50_ of up to 2500 mg/kg b. wt.). Compounds with a LD_50 _over 50 mg/kg b. wt. are safe thus DS may be introduced as a highly safe natural medicament (Buck et al., 1976 ▶).

**Table 1:** An overview on modern and traditional aspects of flixweed (Khorasani, 2001; Tavakoli et al., 2012; Li et al., 2010; Mirheidar, 2005; Sun et al., 2004; Sun et al., 2006; Sun et al., 2005; Wang et al., 2004; Zhongfeng, 2007; Maraghi & and Torfi, 2002; Ayoobi et al., 2013; Mohamed & and Mahrous, 2009; Mirzaei et al., 2011; Lee et al., 2013; Pasalar et al., 2013; Mohammadinia et al., 2012

**Constituents of flixweed**	**Pharmacological activity **	**Uses in TPM**
**Fatty acid**	Anti parasite	Cardiotonic
**Oleic acid, linolenic acid, linoleic acid, palmitic acid, stearic acid**	Anti diarrheal	Purgative
**Monoterpenes and sesquiterpenes**	Emollient	Febrifuge
**cis-β-ocimene, longicyclene, menthol**	Laxative	Antipruritic
**neoisomenthyl acetate, alloaromadendrene**	Antipyretic	Antihelmintic
**Volatile oils **	Anti-Inflammatory	Expectorant Astringent
**Benzyl, allyl, propenyl-isothiocyanate and allyl disulfide constituents**	Analgesic	Litholytic
**Cardiac glycoside**	Antioxidant	Aphrodisiac
**Flavonoids**	Cytotoxic	Appetizing
**Quercetine, kaempferol, isorhamnetine**	Anti cancer	Digestive tonic
**Coumarins**		Laxative
**Scopoletine, Scopoline, Xanthtoxol, isoscopoline, psoralene, xanthtoxin**		Skin lightening
**bergaptane**		Antihemorrhoid
**Phenols**		
**Lactones **		
**Descurainolide A and B **		
**Elements **		
**Iron, zinc, manganese, copper**		

Due to the reports in traditional manuscripts of Persian medicine, DS is considered as an effective therapy for the patients suffering from constipation and hemorrhoid (Ahmadieh, 2007 ▶). Arzani noted in his handbook of medicine for beginners, *Mizan al-tibb*, that *khafaqan* (palpitation) may be the result of abundance of black bile and certain medicinal herbs may evacuate the black bile out of body and thus relieve palpitation (Arzani, 1870 ▶). DS is also traditionally defined as a helpful remedy in patients complaining from heartburn due to the abundance in the black bile. Arzani believed that the secretion of *soada* (black bile) can cause feeling of hunger (Arzani, 1915 ▶). In the abundance of *soada* or disturbance in the function of the pancreas, the secretion of *soada* increases abnormally and the patients feel heartburn (*barsooz*) and likes to eat more frequently to relief the pain (Ahmadieh, 2007 ▶). For these patients, a maceration of DS seed in water was highly recommended for the relief of heartburn as can evacuate the *soada* out of the body.

*Descurainia sophia* Webb ex Prantl is known as a safe herbal medicine and is commonly used by people in Iran. Seeds have also reported to have many therapeutic advantages by TPM resources. Despite broad administration of this herb in folk and traditional medicine, there are only two clinical trials assessing its pharmacological activities. Conducting clinical trials with well methodological set to investigate the medical benefits and safety of the DS in long-term use can help to prove the widely uses of this cheap and available medicinal herbs in public.
